# From Dysfunction to Differentiation: A New Paradigm of T Cell Exhaustion

**DOI:** 10.32607/actanaturae.27873

**Published:** 2026

**Authors:** V. M. Stepanova, A. S. Logvinov, E. A. Emelyanov, N. A. Katin, Yu. P. Rubtsov

**Affiliations:** Shemyakin-Ovchinnikov Institute of Bioorganic Chemistry, Russian Academy of Sciences, Moscow, 117997 Russia; Vitebsk Regional Clinical Oncology Dispensary, Vitebsk, 210603 Belarus

**Keywords:** functional exhaustion, T cells, transcription factors, CD8^+^ T cells, cellular immunotherapy, immune checkpoint blockade, negative co-stimulation

## Abstract

CD8^+^ T cell exhaustion is the loss of certain functions typical of cytotoxic lymphocytes. CD8^+^ cytotoxic T cells originate from naive CD8^+^ T cells upon antigen exposure. Chronic or excessive antigen exposure during certain infections and cancers triggers inhibitory mechanisms that lead to functional exhaustion - a loss of the ability to secrete IFN-γ and other cytokines, as well as to produce granzyme- and perforin-containing lytic granules in response to stimulation. Functional exhaustion also affects CD4^+^ helper T cells, which further reduces CD8^+^ T cell cytotoxicity. This leads to the loss of the CD8^+^ T cell’s ability to kill target cells, which negatively impacts immunity and reduces the efficacy of therapeutic T cells. Currently, T cell exhaustion is considered as a multistage, partially reversible differentiation program. Heterogeneous subsets of stem-like progenitor exhausted T cells (Tex-prog) and terminally exhausted T cells (Tex-term) have been identified. These cells differ in their degrees of dysfunction and, thus, in their potential to restore lost functions. The transcriptional exhaustion program is controlled by key transcription factors, such as TCF1, TOX, T-bet, EOMES, BATF, IRF4, etc. Understanding the dynamic changes taking place in the transcriptional profile and cellular phenotype is critical for effective immunotherapy, in particular in immune checkpoint blockade (ICB) and CAR-T cell therapy, because its success depends entirely on the T cell state. In this review, we discuss current concepts on the molecular and cellular mechanisms of T cell exhaustion, various exhaustion-associated phenotypic and functional changes, and opportunities for preventing T cell exhaustion to optimize anti-tumor immunotherapy strategies.

## INTRODUCTION

Functional T cell exhaustion is a programmed process that emerged during evolution as a mechanism to prevent excessive immune activation leading to autoimmunity and lymphoproliferation. It is accompanied by numerous transcriptional and epigenetic rearrangements largely similar to the mechanisms of central T cell tolerance in the thymus and is characterized by the progressive upregulation of immune checkpoint molecules. Due to advances in transcriptomics and proteomics, T cell exhaustion is now believed not to result from chronic antigen exposure, but to be a programmed component of the immune response, involving multi-stage functional differentiation that gives rise to subsets with varying degrees of dysfunction and potential for functional recovery.

In this review, we discuss current concepts of the mechanisms of T cell exhaustion, which are based on the classification of exhausted T cells according to their functional and transcriptional profiles, focusing on key differentiation regulators, in particular transcription factors and signaling pathways. Particular attention is paid to the role of immune checkpoints and emerging opportunities for therapeutic modulation of their activity. We address modern approaches to immunotherapy, such as CAR-T cell therapy and checkpoint blockade, in terms of a new model of exhaustion, as well as prospects for their optimization in treating oncological and autoimmune diseases.

## FUNCTIONAL T AND CAR-T CELL EXHAUSTION: ISSUE FEATURES

The loss of T cell functionality can be induced by three distinct states: anergy, senescence, and exhaustion [1]. Anergy is a state of functional lymphocyte unresponsiveness following antigen exposure in which cells remain viable but fail to respond to stimuli for a long time. Anergy is an immune tolerance mechanism that prevents excessive immune responses; it can be reversed by changing conditions, e.g., by antigen removal or IL-2 addition [2, 3]. T cell senescence is irreversible and characterized by telomere shortening, loss of CD28, cell cycle arrest, and accumulation of p16, p21, and p53 proteins associated with aging or replicative senescence, and accompanied by reduced cytotoxicity [4, 5, 6]. In contrast, exhausted T cells appear during chronic antigen exposure, e.g., in cancers or chronic infections, and they are characterized by a gradual decline in cytokine production [7].

The phenomenon of T cell exhaustion was first encountered in patients with chronic viral infections, including HIV, approximately 25 years ago [8]. Up to this day, the canonical model used to study T cell exhaustion continues to involve mice infected with the lymphocytic choriomeningitis virus (LCMV) strains Armstrong and clone 13 [9]. The Armstrong strain [10] causes an acute infection, whereas clone 13 [11] induces a chronic infection resulting in progressive dysfunction and exhaustion of virus-specific CD8^+^ T cells - a phenomenon similar to that observed during chronic human viral infections such as HIV or hepatitis C [12, 13]. Use of this model has expanded our understanding of various states of exhausted T cells, the interactions between exhausted populations, and their response to immune checkpoint blockade (ICB). However, extrapolation of mouse-derived data to the human organism is challenging due to significant differences in immune system organization and function. In particular, this is important for cancer patients, even though numerous studies have demonstrated that tumor-infiltrating CD8^+^ T cells exhibit a functional exhaustion pattern similar to that encountered in chronic infections [14]. Nevertheless, the transcriptional profiles of CD8^+^ T cells in cancer and viral infections do differ. This difference was demonstrated in a hepatocellular carcinoma (HCC) model expressing the oncogenic SV40 viral protein, which is likely attributable to the persistent presence of antigen and the influence of a highly immunosuppressive tumor microenvironment [15].

Exhaustion of T cells, chimeric antigen receptor-modified T (CAR-T) cells, or T cell receptor-modified T (TCR-T) cells is characterized by elevated expression of immune checkpoints on the cell surface and gradual loss of effector functions. Exhaustion is a natural mechanism used by T cells to avoid hyperactivation and does not necessarily lead to their rapid death. Exhausted cells can remain viable *in vivo* for a long time while continuing to accumulate molecular and functional changes that reduce the efficacy of adoptive immunotherapy [14].

Overwrought or prolonged T cell activation, whether via the T cell receptor (TCR) or CAR, is considered the primary cause of exhaustion. In the case of CAR-T cells, there is a tonic signaling effect where antigen-independent CAR activation, driven by the receptor’s structure, triggers, by itself, the early stages of exhaustion [16]. Of similar significance is the tumor microenvironment that affects T cells via inhibitory receptors. Tumor cells express ligands for inhibitory receptors, such as PD-L1 [17], and secrete molecules and exosomes that suppress CAR-T cell activity [17, 18]. In addition, myeloid cells and regulatory T cells suppressing the immune response are present in the tumor microenvironment [19]. An important participant in this process is tumor-associated macrophages that are one of the major sources of antigen stimulation, resulting in terminal T cell exhaustion [20, 21]. Clinical studies have demonstrated that the degree of CAR-T cell exhaustion correlates with therapeutic efficacy and outcome: non-responders have higher counts of exhausted T cells. This underscores the importance of developing strategies for overcoming exhaustion to enhance the sustainability of the therapeutic response.

**Exhausted T cells are a distinct subset of CD8^+^ cells**

The differentiation of T cells from a naive state into effector and memory cells is a well-characterized process that is accompanied by distinct transcriptional, metabolic, and functional changes. Exhausted T cells have primarily been studied in the CD8^+^ T cell population, whereas CD4^+^ T cells are also susceptible to exhaustion [22, 23]. However, there is no clear understanding of the mechanisms underlying the development of exhausted CD4^+^ lymphocyte populations and the factors regulating the switch from a normal to exhausted phenotype.

Progenitor exhausted T cells (Tpex) were previously believed to form exclusively during chronic infections, cancers, or autoimmune diseases; i.e., upon prolonged antigen exposure [24]. However, new data indicates that these progenitors can also arise at the early stages of an acute infection, even when the infection is successfully controlled by the immune system [12]. This challenges the previous paradigm holding that exhaustion results solely from chronic antigen exposure. In other words, exhaustion is a programmed component of the immune response, which is activated earlier than it was previously thought, already at the stage of early exhausted effector cells [12]. Concepts regarding the differentiation direction of exhausted T cells also evolve. Earlier, differentiation of these cells was thought to be linear, similar to that of conventional T cells. Currently, there is growing evidence indicating branched trajectories in the development of exhausted cells; thus, they may be classified as a distinct CD8^+^ T cell subset (*Fig. 1*) [25, 26]. This view is supported by studies of the TCR repertoire in Tex cells, which is as diverse as that of naive T cells, unlike the high clonality of TEMRA. Although prolonged and robust TCR-mediated activation is undoubtedly the primary driver of exhaustion, the transcription factors T-bet and EOMES also play a significant role in this process. Under normal conditions, during the first division following TCR/MHC-mediated activation, a naive CD8^+^ T cell divides asymmetrically: one daughter cell receives more T-bet and becomes an SLEC (effector cell), whereas the other receives more EOMES and transforms to an MPEC (memory precursor) [27, 28]. Upon exhaustion, the pattern changes drastically: both factors, T-bet and EOMES, act in concert [29]. They do not just regulate the balance between effector and memory cell, but they participate in the formation of a distinct lineage - exhausted T cells (Tex) [29, 30, 31]. This further confirms that Tex is a separate T cell developmental lineage that is different from the classical SLEC/MPEC phenotypes.

**Fig. 1:**
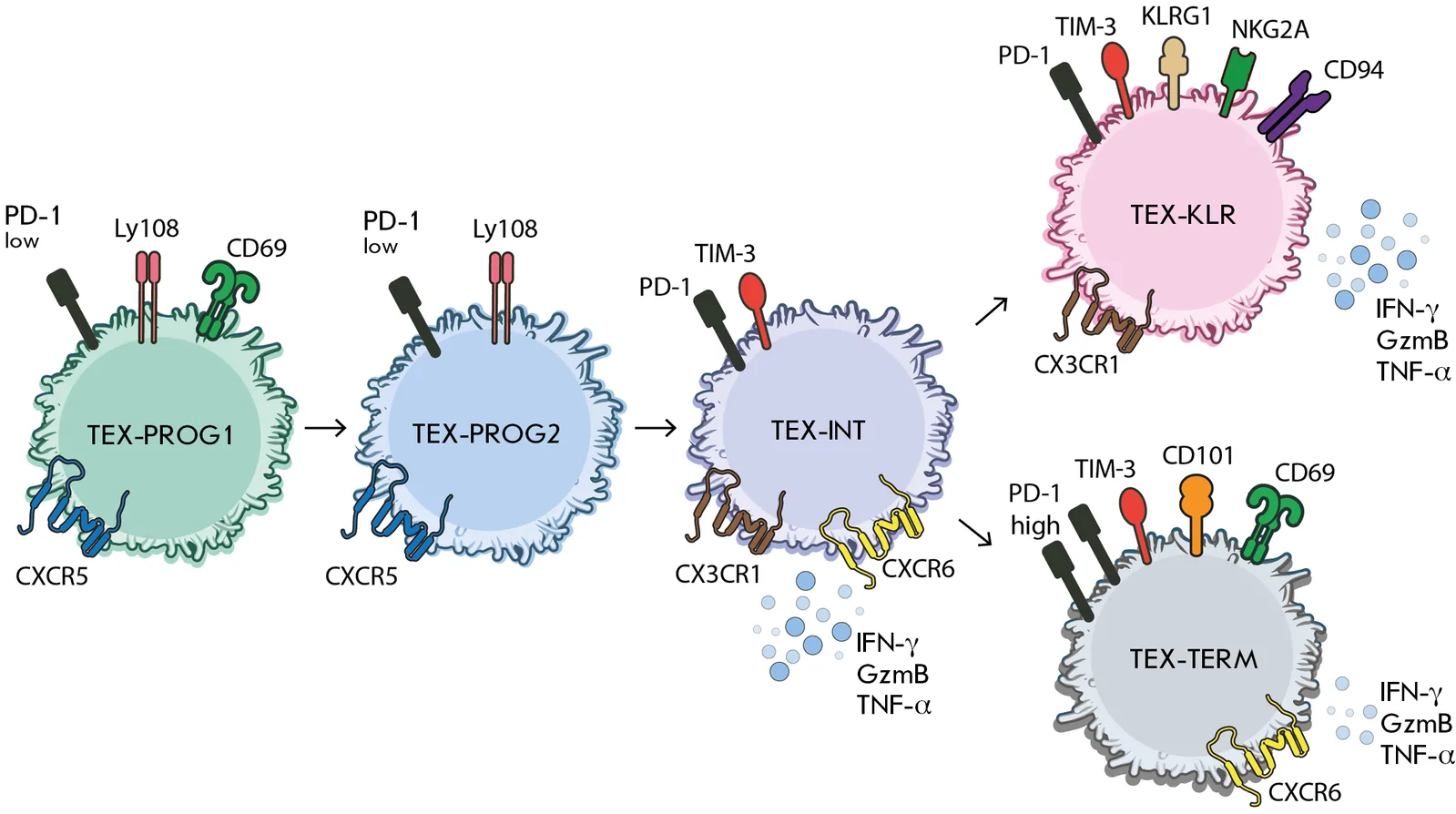
Surface markers characteristic of exhausted CD8^+^ T cell subsets. Schematic representation of the phenotypic features of major exhausted CD8^+^ T cell (Tex) subsets: stem-like Tex-prog1 (resident) and Tex-prog2 (circulating) progenitors, intermediate (Tex-int), terminally exhausted (Tex-term), and NK-like (Tex-KLR) populations. The key surface markers associated with each population are indicated, in particular PD-1, TIM-3, CXCR5, CX3CR1, CD101, KLRG1, etc. These markers enable discrimination of populations based on their differentiation stage, functional activity, and tissue localization

**Trajectory of T cell exhaustion**

Chronic TCR-mediated antigen stimulation, which is associated with NFAT pathway activation without the involvement of the AP-1 factor, triggers the program of naive CD8^+^ T cell differentiation into exhausted cell progenitors. These cells, known as stem-like resident progenitor exhausted T cells (Tex-prog), possess the ability for self-renewal and proliferation as well as a phenotype similar to that of central memory cells (CCR7^+^CD62L^+^) [32, 33]. Tex-prog cells experience a multipotent fate; their further differentiation is controlled by the pattern of microenvironmental signals: they either proliferate while retaining stem-like properties, or differentiate into intermediate exhausted cells (Tex-int), or transit directly to a terminally exhausted state (Tex-term). The key role in maintaining the Tex-prog pool is played by cytokine IL-15, which promotes proliferation of these cells [34].

An earlier population of precursor exhausted T cells, termed Ex-Pre cells, were recently identified. Under the influence of the stress-induced transcription factor Btg1, these cells can differentiate into Tex-prog cells that form the pool of exhausted cells [12]. Under certain conditions, Tex-prog cells, facing a multimodal fate, can re-differentiate into cells with a central memory phenotype or, conversely, get activated and acquire effector functions, which is apparently regulated by the strength of TCR-mediated signaling, as well as via the STAT5 and mTOR signaling pathways [35, 36] (*Fig. 2*). In a chronic LCMV infection, Raposo et al. demonstrated that some exhausted CD8^+^ T cells with the Tex-cm phenotype (exhausted memory cells) retained traits of central memory cells and generated a protective immune response upon re-exposure to the antigen [37]. Due to their self-renewal ability and potential for functional recovery, Tex-prog cells serve as a key target for immunotherapy, particularly for immune checkpoint inhibitors.

**Fig. 2:**
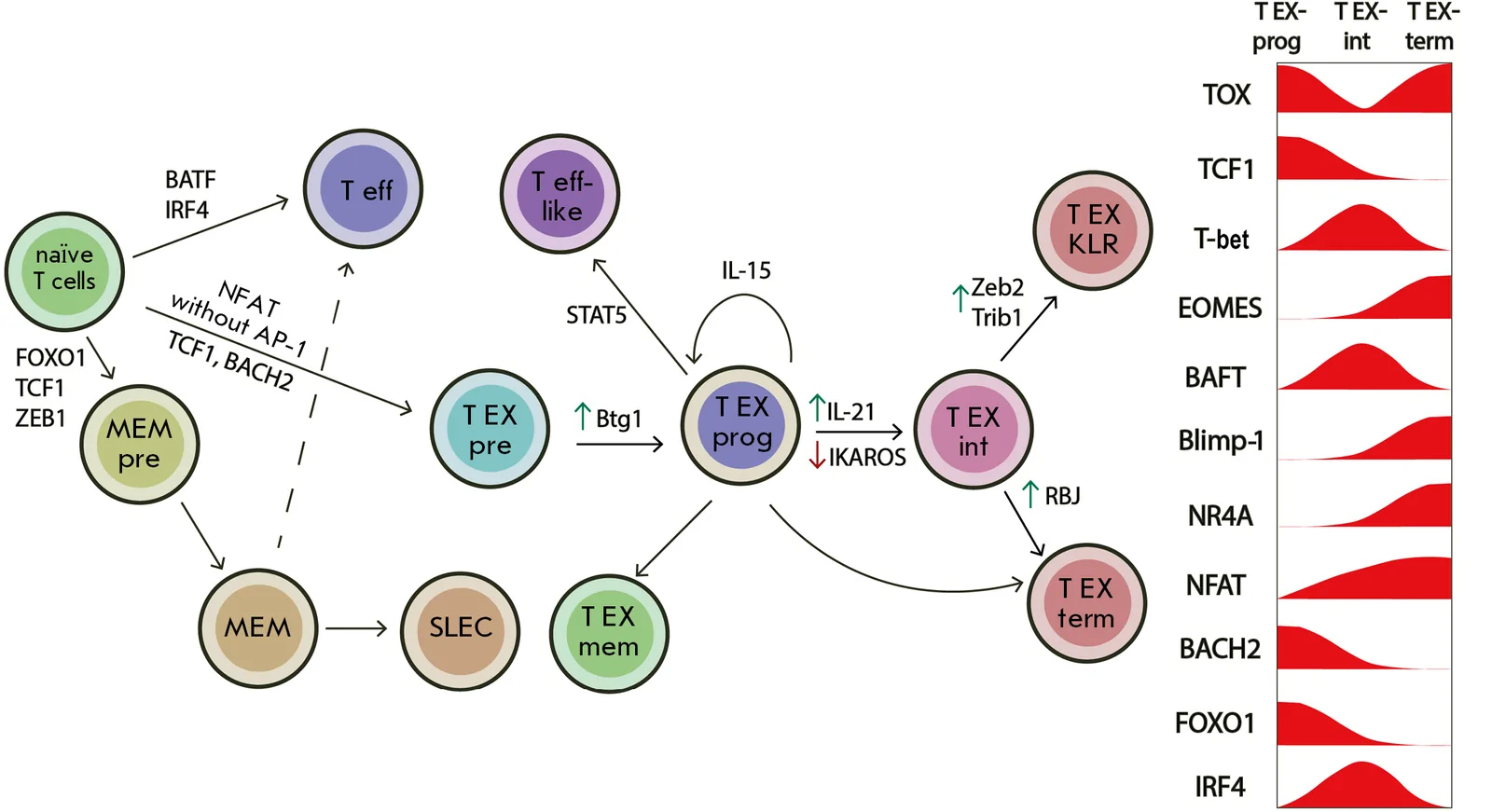
Differentiation trajectory of exhausted CD8^+^ T cells. Stages of differentiation of naive CD8^+^ T cells into exhausted T cells (Tex) during a chronic antigen stimulation. Key transitions are shown: from naive T cells to early exhaustion precursors (Ex-Pre), followed by stem-like progenitors (Tex-prog1 and Tex-prog2), intermediate (Tex-int), and terminal (Tex-term and Tex-KLR) populations. Expression of the characteristic transcription factors TCF1, TOX, T-bet, EOMES, etc. is indicated for each stage (right panel). Differentiation is regulated by TCR signal strength, cytokine microenvironment, and expression of inhibitory receptors. Overview of the major transcriptional regulators involved at various stages of exhausted phenotype formation. The dynamics of transcription factor expression (TCF1, TOX, T-bet, EOMES, BATF, IRF4, FOXO1, ID3, NR4A, Zeb2, etc) across differentiation stages is depicted – from stem-like progenitors (Tex-prog) to terminally exhausted (Tex-term) and NK-like (Tex-KLR) cells

Almost all exhausted CD8^+^ Tex subsets are PD-1-positive, with PD-1 levels progressively increasing as exhaustion develops. Tex-prog cells are characterized by low PD-1 levels, and their phenotype is similar to that of CD4^+^ follicular helper T cells (Tfh) [38]. Studies exploiting a mouse model of a chronic LCMV infection have shown that Tex-prog cells express markers such as TCF-1, CXCR5, Ly108, CD73, and ID3, as well as low levels of CD127 and CD62L; however, they lack expression of the inhibitory receptor TIM-3 [39, 40]. Tex-prog cells also express CCR7, which enables them to respond to CCL19 and CCL21 − chemokines secreted by reticular fibroblasts in the paracortex of lymph nodes [39]. This fact is indication that TCF1^+^ Tex cells can actively migrate into lymphoid structures, where they persist and actively proliferate. Furthermore, TCF1^+^ stem-like Tex-prog cells express CXCR5, which may facilitate their migration in response to CXCL13 secreted in lymphoid structures. Although the CXCR5 function in the context of cancer immunology has not been fully elucidated, there is evidence that it may be involved in the localization of stem-like CD8^+^ Tex cells in the tumor, in particular in tertiary lymphoid structures (TLS). These findings were reported in studies on non-small cell lung cancer (NSCLC) where CXCR5^+^ T cells were identified in the tumor and associated with TLS [41, 42]. Histological analysis of prostate, bladder, and kidney cancer biopsies revealed that TCF1^+^CD8^+^ T cells were predominantly located in dense MHC II regions, whereas terminally exhausted TCF1^-^CD8^+^ TILs were diffusively distributed [43]. These data suggest that dense MHC II niches may play a crucial role in maintaining stem-like properties and recruiting Tex cells, probably through interactions with dendritic cells and the Wnt signaling pathway that supports TCF1 expression.

Tex-prog cells may be functionally classified into two subsets: resident Ly108^+^CD69^+^TCF1^+^ located in secondary lymphoid organs (Tex-prog1) and circulating Ly108^+^CD69^-^TCF1^+^ (Tex-prog2) stem-like cells. Unlike Tex-prog1 cells, Tex-prog2 cells are characterized by a high proliferative potential and give rise to an intermediately exhausted population [30, 31]. Systematic cataloging of the repertoire of surface molecules expressed by exhausted CD8^+^ T cells has revealed additional surface molecules with less well-characterized roles in T cell exhaustion, such as CD7, Lax1, CD200R, and CD101 [32, 33] (*Fig. 1*).

Upon exposure to a combination of transcription factors and antigen stimulation, progenitor cells can differentiate either into a population of intermediate CD101^-^TIM-3^+^ exhausted T cells (Tex-int) or directly into CD101^+^TIM-3^+^ terminally exhausted cells (Tex-term) [33]. The intermediate population (Tex-int) is characterized by elevated expression of the transcription factor T-bet and the migration and activation marker CX3CR1. The chemokine receptor CX3CR1 is required for cell migration into the splenic red pulp and the execution of tissue-specific effector functions. Tex-int cells lose the Ly108 marker but express a set of exhaustion-associated molecules, including CXCR6, TIM-3, and Klf2 [34]. Another important marker of Tex-int cells is CXCR3. It interacts with CXCL10 secreted by fibroblasts in response to IFN-γ, particularly during a chronic infection or immune checkpoint blockade (ICB) therapy. Binding to CXCL10 can reduce TCF-1 expression and promote the differentiation of Tex cells into effector cells. Tex-int cells exhibit low cytotoxic activity and continue to circulate in the bloodstream, which may be indication of their intermediate position between Tex-prog and Tex-term cells in differentiation [30, 44]. The formation of this population is highly dependent on IL-12-secreting CD4^+^ T cells and the histone deacetylase HDAC1 [45], which stimulate the transition from Tex-prog to Tex-int [32].

Two existing terminal populations − Tex-term (terminally exhausted T cells) and Tex-KLR − are no longer plastic enough and cannot revert to either a stem-like or intermediate state. This final transition is coordinated by the antagonism of TOX and EOMES against T-bet [29]. However, while exhaustion was previously thought to progress linearly, a new theory proposes that terminal exhaustion develops along three possible trajectories: a Tex-term bifurcation trajectory, a Tex-KLR bifurcation trajectory, or a mixed trajectory where the Tex-int population serves as the bifurcation point [35]. The choice of trajectory correlates with TCR signal strength, antigen density, and the location of a certain Tex clone [35, 44]. Clones with high-affinity TCRs are predisposed to the Tex-term fate, whereas clones with low-affinity TCRs transit into Tex-KLR cells. When Tex-int cells migrate into several organs, their differentiation trajectories are maintained; however, Tex-KLR-prone clones are rarely found in the liver, unlike Tex-term clones, which may be indication of their phenotypic adaptation to the specific tissue microenvironment [37, 46]. Although both exhausted populations originate from Tex-int cells, they differ drastically in phenotype and function. Like Tex-int cells, Tex-KLR cells express CX3CR1, PD-1, and TOX, but at lower levels, and more closely resemble effector T cells than their precursors [46, 47]. These cells are characterized by high levels of KLRG1, in addition to other C-type lectin-like receptors (KLRs), such as NKG2D, NKG2A, CD94, and NK1.1. Granzymes and ZEB2, T-bet, and ID2 transcription factors, expressed by both effector T cells and natural killer (NK) cells, are also typical of Tex-KLR cells [34, 48]. High cytotoxicity and low CXCR6 levels distinguish Tex-KLR cells from the Tex-term population that exhibits elevated expression of exhaustion markers (CD101^+^, CD69^+^, TIM-3^+^) and other checkpoint receptors, such as LAG-3, TIGIT, etc. [35]. Tex-KLR cells are functionally active compared to Tex-term cells, and increasing the proportion of Tex-KLR cells enhances therapeutic efficacy under chronic antigen exposure. Thus, there is significant interest in the search for strategies to modulate the transition from the Tex-int to the Tex-KLR state in situations where exhaustion cannot be prevented. Anti-PD-L1 ICB alone cannot redirect cells to differentiate into Tex-KLR cells, but a combination of anti-PD-L1 immunotherapy and knockout of the pseudokinase Trib1 yields sustained accumulation of effector-like Tex-KLR CD8^+^ T cells [49, 50].

At the moment, this classification provides better insight into the exhaustion trajectory; however, the characterized populations remain heterogeneous, which suggests the need for further expanding exhaustion phenotypes in order to better understand the exhaustion process as a whole.

**CD4^+^ T cells: regulators of or participants in exhaustion?**

CD4^+^ T cells facilitate the development of a more robust and functionally active CD8^+^ T cell response, particularly during a chronic infection. Although CD4^+^ T cells are not strictly required for the emergence of TCF1^+^ progenitor exhausted CD8^+^ T cells, CD4^+^ T cells are critical for their differentiation into effector subsets and for maintaining a pool of cells capable of proliferating and responding to immunotherapy.

While CD8^+^ T cell exhaustion has been explored in detail, information on exhausted CD4^+^ T cells remains limited due to their functional diversity, plasticity, and differentiation complexity, which hinders the clear definition of a universal exhausted phenotype. Attempts to identify exhaustion in CD4^+^ cells have confirmed that, like CD8^+^ T cells, CD4^+^ cells can enhance the expression of common inhibitory receptors [51]. Also, upon prolonged antigen exposure, CD4^+^ T cells were found to lose their functional activity, but to recover upon antigen removal [23].

As with CD8^+^ cells, distinct populations of exhausted CD4^+^ cells can be identified (*Fig. 3*). Clonal fate analysis of T cells and experiments on their adoptive transfer revealed that PD-1^+^TCF1^+^CD4^+^ cells with a memory-like phenotype, similar to TCF1^+^ CD8^+^ cells, possess self-renewal ability and give rise to both Teff and TFH cells, thereby functioning as exhausted cell progenitors [52]. The development of these progenitors depends on *Bcl6*, and they are required for maintaining the antigen-specific CD4^+^ T cell response during a chronic infection [53]. Virus-specific TCF1^+^BCL6^+^CD4^+^ T cells are also capable of differentiating into Th1 or TFH cells. For example, tumor-associated TFH-like cytotoxic CD4^+^ T cells are characterized by high LAG-3/Blimp-1 expression and low TCF1 expression and lack self-renewal ability, whereas neurotoxic TFH cells express low levels of LAG-3/Blimp-1 and high levels of TCF1 and retain self-renewal ability. This pattern closely tracks the relationship between terminally differentiated cells and stem-like progenitors upon CD8^+^ T cell exhaustion [50, 52, 53].

**Fig. 3:**
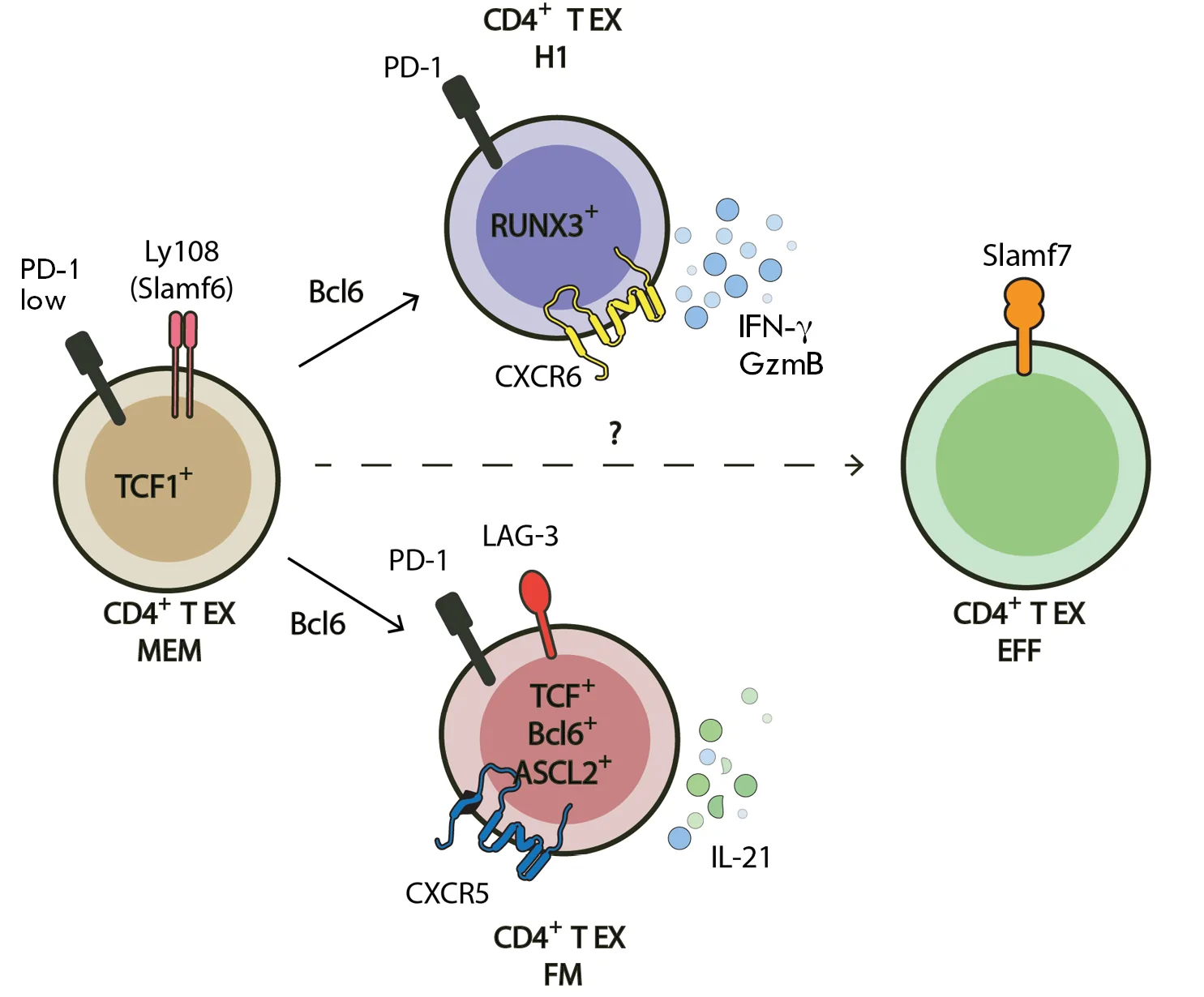
Surface markers characteristic of exhausted CD4^+^ T cell subsets. Phenotypic heterogeneity of exhausted CD4^+^ T cells upon chronic antigen exposure. The main subsets are shown: TCF1^+^ progenitors with self-renewal potential, Th1-like and TFH-like effector cells, as well as Slamf6^+^ and Slamf7^+^ populations exhibiting memory- and cytotoxicity-associated features, respectively. Key markers are indicated, including CXCR5, CXCR6, PD-1, LAG-3, Blimp-1, and Slam family proteins, which enable discrimination of the functional states of CD4^+^ T cells in the context of exhaustion

The CD4^+^ T cell population has been found to consist of three transcriptionally and epigenetically distinct subsets: CXCR6^+^CXCR5^-^ Th1 cells, CXCR5^+^CXCR6^-^ Tfh cells, and a Slamf6^+^(Ly108) subset exhibiting memory-like traits (Tmem, memory-like CXCR5^-^CXCR6^-^). On the basis of the transcription of some genes, the Tmem subset resembles memory cells; these cells express cytokines and effector molecules at low levels and possess limited proliferative potential [54]. These long-lived cells accumulated during a chronic infection or following repeated antigen exposure [32]. The existence of transcriptionally distinct CD4^+^ cell exhaustion states that develop at the peak of infection has been confirmed, and a previously unknown Slamf7^+^ subset exhibiting cytotoxic properties has been identified [55, 56]. However, it remains unclear which factors drive the differentiation of CD4^+^ precursors into a certain subset. Probably, as in CD8^+^ cells, TCR affinity plays an important role because the strength of the TCR signal is negatively correlated with Th1 cell formation during a chronic infection. However, this is likely not the primary factor behind differentiation, as clones expressing the same TCR can acquire either a Th1 or TFH phenotype. Additional signals, such as specific cytokines and TCR stimulation or co-stimulation rates, may also be involved in this process.

**Transcriptional regulation of exhaustion**

T cell exhaustion is a complex, multimodal process that is not only driven by a set of checkpoints but is also regulated by the key transcription factors involved in establishing and maintaining the exhausted state of CD8^+^ T cells [57]. Although persistent antigen stimulation via the TCR and activation of the transcription factor NFAT are required to initiate and stabilize the exhausted state, the interplay of specific transcriptional regulators is what provides the stability and reversibility to this process [58]. Some of these, such as TOX [59] and TCF1 [60], may be considered key regulators, whereas others (T-bet and EOMES [31], FOXO1 [61], EGR2, IRF4 [62, 63], NR4A2, and recently identified MYB [64], BATF [57], ID3 [65, 66], BTG1 [49], IKAROS [67], ETS1 [67], RBPJ–IRF1 [67], ZEB2 [49], and Trib2 [50]) coordinate transcriptional rearrangement at various stages of exhaustion (*Fig. 4*). Despite significant progress in deciphering these mechanisms, many aspects remain insufficiently understood, particularly within the context of the tumor microenvironment and the dynamic changes in response to immunotherapy.

**Fig. 4:**
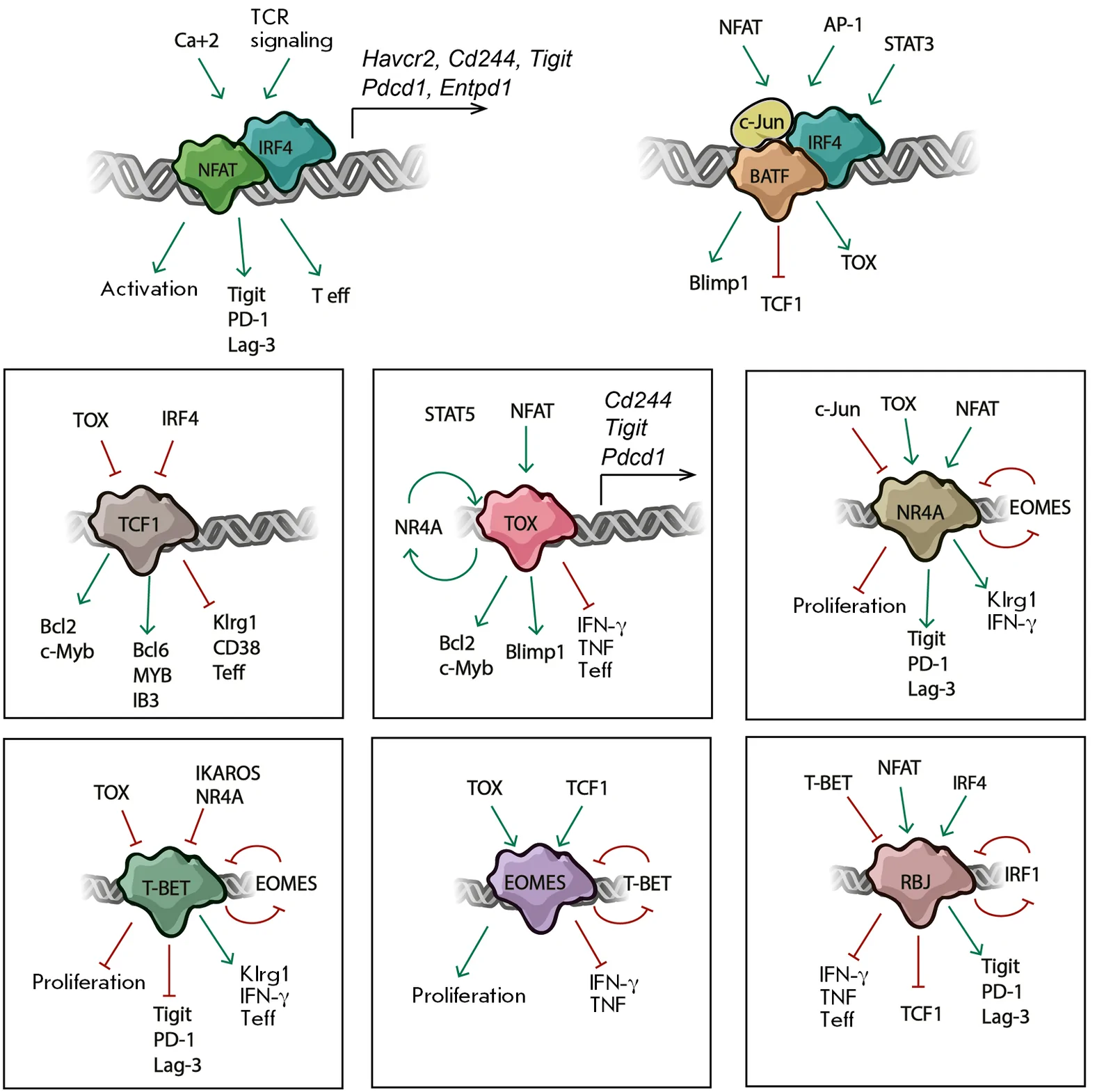
Key transcription factors regulating CD8^+^ T cells exhaustion. Mutual regulation of the transcription of genes encoding transcription factors (targets during T cell exhaustion) and major non-transcriptional regulatory targets. Signaling hierarchy and principal drivers of transcriptomic changes during the development of the exhausted phenotype are shown. Transcription factors and other proteins are depicted schematically

**TCF1**

TCF1 (*TCF7*) is one of the key regulators of stemness in exhausted T cells [68]. This factor is highly expressed upon BATF and NFAT activation [58] in stem-like progenitor exhausted T cells (Tex-prog) and is virtually absent in terminally exhausted cells (Tex-term) [59, 60]. TCF1 is a key transcription factor required for the maintenance of Tpex cells; it regulates the transition between terminal effector cells and Tpex cells by suppressing the former and stabilizing the latter [69]. This stabilizing function of Tpex cells is implemented through the upregulation of EOMES, FOXO1, ID3, and MYB, which counteract differentiation by suppressing expression of the genes associated with terminal exhaustion, such as Blimp-1 (*Prdm1*) and TOX [24], and potentially promote cell survival by maintaining Bcl-2 expression [60]. At the same time, TCF1 regulates the expression of the *Bcl6* gene that promotes a phenotype resembling that of progenitor cells and facilitates the generation of memory cells [69, 70].

TCF1 knockout in T cells reduces the number of exhausted CXCR5^+^PD-1^+^ T cells but simultaneously diminishes T cell persistence during a chronic viral infection [71, 72]. The regulation of TCF1 expression is closely tied to the activity of the transcription factor FOXO1 [73]. FOXO1 activity is inhibited via AKT-mediated phosphorylation in response to TCR activation, which is indication of a relationship between TCR signaling and restriction of TCF1 expression. Interestingly, high levels of PD-1 expression during the early stages of a chronic infection are required to maintain the TCF1^+^ population of progenitor exhausted T cells [74], and constriction of PD-1-mediated TCR signaling may facilitate FOXO1 activation, thereby enhancing TCF1 expression, although this mechanism requires further investigation.

**TOX and NR4A**

The transcription factor TOX plays a key role in the regulation of T cell exhaustion. It not only controls this process but also is found in all activated CD8^+^ T lymphocytes, particularly in memory cell subsets, including effector memory T cells and terminally differentiated effector memory T cells re-expressing CD45RA (TEMRA) [75]. TOX expression is also characteristic of Tex-prog cells; it decreases in intermediate populations and increases again upon prolonged NFAT activation [58] (*Fig. 3*). TOX promotes epigenetic fixation of the exhausted phenotype by triggering an irreversible exhaustion process, upregulating inhibitory receptors such as PD-1, TIM-3, TIGIT, and LAG-3, and suppressing the transcription factor T-bet. Seo et al. demonstrated that tumor-specific TOX- and TOX2-deficient CAR-T cells exhibit reduced levels of inhibitory receptors and an enhanced anti-tumor response [76].

The process of T cell exhaustion is also regulated by several transcription factors, including NR4A1 and NR4A2, which act synergistically with TOX [58, 59, 76, 77] (*Fig. 4*). In the context of the anti-tumor immune response, NR4A deficiency in CAR CD8^+^ T cells was shown to be accompanied by reduced TIM-3 expression and increased production of TNF and IFN-γ. The genomic profile of NR4A-deficient cells corresponds to an effector T cell phenotype, as evidenced by low TCF1 levels [78]. Cytokine signaling pathways also play a crucial role in this regulation: interleukin-2 receptor (IL-2R)-mediated signaling promotes differentiation into Tex cells at the expense of the Tex-prog population [79], whereas activation of IL-21R-mediated signaling at later stages stimulates the development of Tex-prog cells [80]. Another key regulator is STAT5, which can suppress TOX expression to prevent T cell exhaustion [81]. Elevated STAT5 expression increases the number of Tex-int cells and promotes the formation of stable effector cells exhibiting high antitumor activity.

**T-bet/EOMES and IRF4/BATF**

The transcription factors T-bet and EOMES play a key role in maintaining CD8^+^ T cell effector function. Their interaction specifies the direction of T cell differentiation [82]. A predominance of T-bet leads to more pronounced terminal exhaustion, whereas EOMES dominates in the memory cell population [83]. The nuclear EOMES-to-T-bet ratio is elevated in Tex cells compared to that in memory cells [29, 81]. Forced nuclear localization of T-bet in Tex cells induces differentiation resembling that of effector cells [74].

T cell exhaustion is also regulated by the transcription factors BATF and IRF4. These factors enhance TCR signaling and regulate T cell differentiation, facilitating the transition from Tex-prog cells to an intermediate exhausted population [62, 63, 84]. Overexpression of BATF in murine CAR-T cells promotes its preferential interaction with IRF4, which redirects the T cell transcriptional program away from exhaustion towards effector function and memory [85]. Similar results were reported for the overexpression of c-Jun, a transcriptional partner of BATF and IRF4, in human CAR-T cells [86].

In contrast to BATF/IRF4, the factor BACH2 is highly expressed in Tex-prog cells [8, 87, 88]. BACH2 deficiency in CD8^+^ T cells impairs the formation of early Tex-prog cells, whereas its overexpression promotes the development of stem-like exhausted cell progenitors [88] and inhibits the function of IRF4, Blimp-1, and AP-1 family members (including BATF) [8, 88]. During the late stages of a chronic LCMV infection, activity of BATF, and to some extent that of T-bet, induces differentiation of Tex-prog cells into a CX3CR1^+^ effector-like population [88].

**Other regulators and their functions**

Genetic and functional studies have identified other key regulatory factors governing the differentiation of exhausted T cells: IKAROS and ETS1. Together, they regulate the exit of cells from the quiescent state (the state of Tex-prog1) and the transition of Tex-prog2 cells to the Tex-int state [89]. IKAROS prevents Tex-prog1 activation by acting through TCF1; its deficiency enhances Tex-prog2 accumulation, but it suppresses their further differentiation [89]. In contrast, ETS1 acts via BATF and promotes the transition of Tex-prog cells to a more mature state [67, 90].

There is another RBJ–IRF1 factor combination that acts as a regulator of terminal exhaustion. RBJ controls the transition from Tex-int to Tex-term cells, particularly in response to IFN-γ and Notch signaling [91]. It prevents terminal exhaustion, preserves the proliferative and functional potential of cells, and enhances the response to ICB [24].

Another universal regulator of T cell stemness is ID3, which acts during acute and chronic infections, as well as neoplastic diseases. During an acute infection, ID3-expressing T cells can form Tex-prog cells whose survival is supported by ID3 through reduced apoptosis susceptibility induced by Fas binding to a Fas ligand. IL-1 family cytokines (including IL-36β and IL-18) also promote the development of ID3^+^ stem-like T cells [66]. The transition from the Tex-int state to the Tex-KLR state is regulated not only by the above-mentioned factors, but also by the pseudokinase Trib2 and transcription factor ZEB2 [50].

The described diversity of transcription factors is just the tip of the iceberg regarding the regulation of CD8^+^ T cell exhaustion. They are all closely interrelated (*Fig. 5*) and form a complex regulatory network that controls the cell’s further fate [92].

**Fig. 5:**
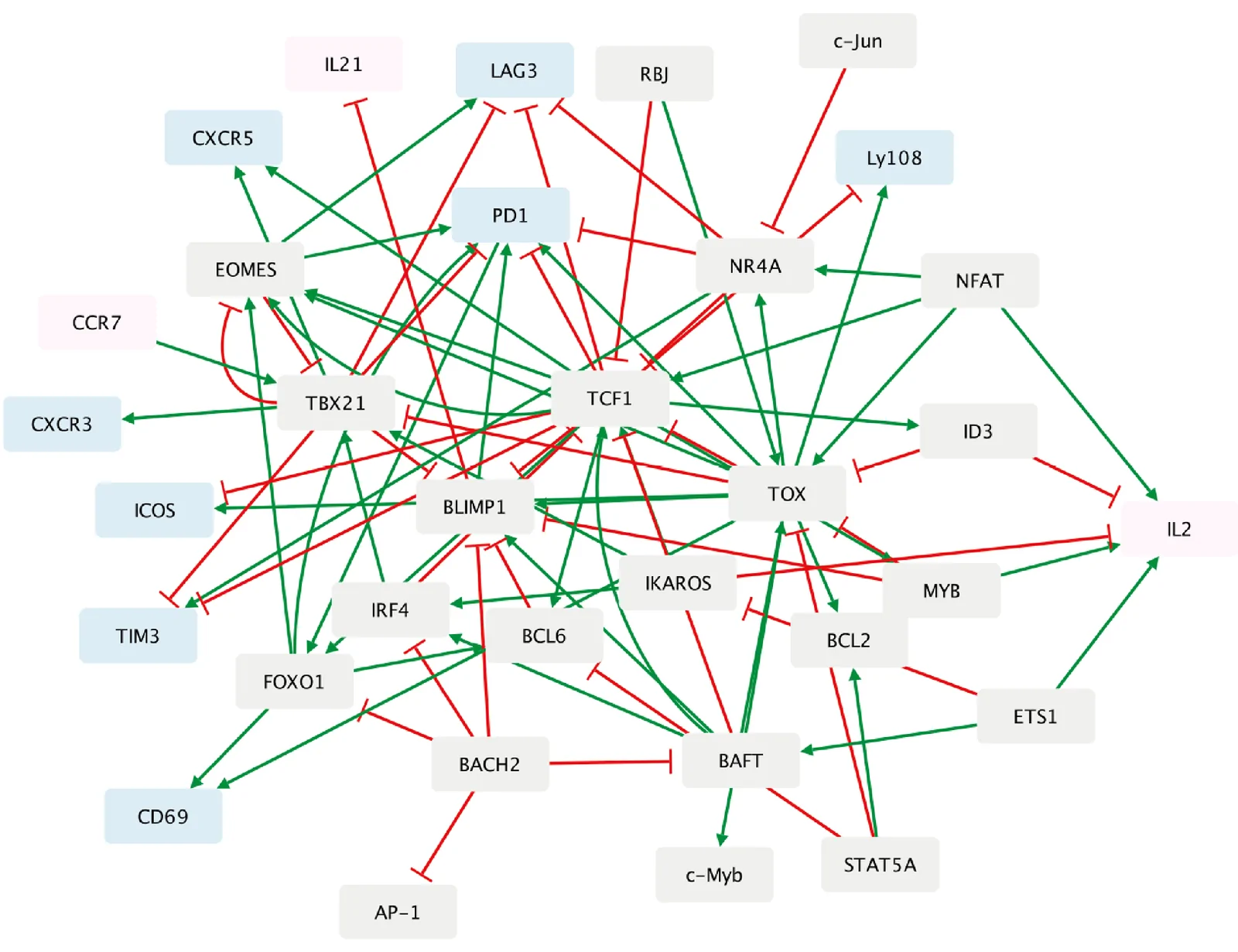
The transcriptional regulatory network of T cell exhaustion. A graphic model of interactions among the key transcription factors involved in the regulation of exhausted T cell differentiation. The network generated using Cytoscape [93] depicts activating (solid arrows) and suppressive (blunt end arrows) interactions between the factors (TCF1, TOX, T-bet, EOMES, BATF, IRF4, FOXO1, RBPJ, IKAROS, ETS1, etc.), based on the transcription factors listed in *Supplementary Table 1*. This regulatory network determines a cell’s fate by guiding cells along differentiation trajectories and ensuring response plasticity during chronic antigen exposure

**Immune checkpoints**

To date, a significant number of inhibitory molecules, known as immune checkpoints, which are regulatory proteins acting as watchdogs of the immune system, have been identified. These molecules contain specific signaling domains, such as the tyrosine-based inhibitory motif (ITIM) and the immunoreceptor tyrosine-based switch motif (ITSM), which enable the transmission of inhibitory signals into the cell [94]. The diversity of ligands, including MHC I/II, the B7 family, CD155, HVEM, and galectin-9, as well as the differences in intracellular signaling domains, (ITIM, ITSM) provides the basis for variability in the effects of each checkpoint on T cells.

The involvement of checkpoints in T and CAR-T cell exhaustion may correlate with the stage of T cell differentiation; molecules, such as LAG-3, PD-1, and CTLA-4 [95], are associated with the early stages of exhaustion [96]. TIGIT, CD112R, and BTLA enhance suppression of cellular function at the intermediate exhausted T cell (Tex-int) stage [97, 98], whereas TIM-3 and VISTA are more frequently expressed on terminally exhausted cells [99, 100] (*Fig. 1*).

As previously mentioned, all exhausted CD8^+^ T cell (Tex) subsets express PD-1, to varying degrees. However, under the new conceptual framework, PD-1 is not merely a marker of exhaustion; it reflects distinct stages of exhaustion where the PD-1^+^CD8^+^ Tex is a pool of cells exhibiting varying degrees of exhaustion, from stem-like progenitors (PD-1low) to terminally exhausted cells (PD-1high) [38]. Regardless of PD-1 expression levels, immune checkpoint blockade (ICB) therapy is effective only when Tex-prog cells remain in the body. By acting on these cells, ICB therapy activates their proliferation, thereby increasing the pool of stem-like exhausted T cells and restoring their function [101]. Previously, checkpoint inhibitors were believed to restore the activity of exhausted T cells directly in the tumor [102]. However, recent evidence indicates that exhausted T cells in the tumor often fail to respond to therapy [103]. TCF1^+^CD8^+^Tex-prog cells located in lymph nodes, rather than in the tumor, are the major population responding to ICB therapy [104, 105].

**CAR-T cell exhaustion in terms of a new paradigm**

The expansion of CAR-T therapy beyond oncology into the area of autoimmune diseases, such as systemic lupus erythematosus and multiple sclerosis, has highlighted a common challenge: in both clinical contexts, the persistence and efficacy of modified T cells are limited by the development of an exhaustion phenotype [106, 107]. Deciphering the regulatory networks that control lymphocyte dysfunction under chronic antigen exposure provides the basis for revisiting existing paradigms in cell-based therapy. The new pattern of T cell exhaustion opens up opportunities for improving adoptive cell immunotherapy and immune checkpoint blockade (ICB) therapy.

First, systematic cataloguing of the surface molecule repertoire has led to the development of the Cyclone algorithm that analyzes T cell clone changes over time and identifies their functional states [101]. Furthermore, the identification of novel, differentially expressed markers (CD101, TIM-3, Ly108, Slamf6, and CD69) that characterize distinct exhaustion states will enable the introduction of a new classification of exhaustion to analyze anti-tumor therapy potential and assess ICB applicability [30, 33]. Apart from classical immune checkpoints, CD8^+^ T cell exhaustion can be further supported by two non-classical extracellular mechanisms - phosphatidylserine exposure on the surface of viable lymphocytes, which suppresses dendritic cells; and the thrombospondin-1/CD47 axis that triggers pathogenic calcineurin–NFAT signaling - thereby opening up new opportunities for immunotherapy directed at these targets [108, 109].

Second, several modern therapeutic strategies aim to restore a normal phenotype to exhausted cells; however, it is becoming evident that this approach is not applicable to the entire Tex population. Furthermore, Tex-term cells, which are considered undesirable, may be specifically adapted for survival in niches with high antigen levels. Further research will elucidate whether restoring effector function can preserve these properties of Tex-term cells.

Third, the strength of TCR signaling drives the fate of T cells. Therefore, in addition to antigen recognition, phenotypic outcomes of TCR activation should be evaluated upon developing TCR- and CAR-based cell therapies.

Finally, the understanding that the polyclonal T cell response to chronic antigen exposure strikes a balance between effector and memory-like functions and is implemented via the development of two distinct Tex states suggests that future cell therapies will be tailored to a specific phenotype of patient exhausted T cells. Indeed, molecules characteristic of Tex-KLR cells have recently been identified in exhausted human CAR-T cells [110]. The finding suggests that this cell type may also be present during adoptive cell therapy. Manipulating the gene regulatory programs of Tex states may provide important avenues for developing advanced T cell-based immunotherapies.

Furthermore, approaches that seek to increase the percentage of Tex-prog cells, accelerate their differentiation into Tex-int cells, and prevent their transition to the terminal exhaustion state appear promising. These strategies may become the basis for combination immunotherapy that integrates T cell genetic modifications with transcriptional regulator and signaling pathway manipulations aimed at overcoming exhaustion and enhancing therapeutic efficacy.

## CONCLUSION

T cell exhaustion, initially defined as passive dysfunction, is now understood as an active, regulated differentiation program with specific stages and molecular determinants. The emergence of the concept of heterogeneous exhausted cell subsets, including self-renewing progenitors (Tex-prog) and intermediate or terminally exhausted states (Tex-int, Tex-term, and Tex-KLR), has drastically altered the approach to our interpretation of the immune response and its modulation in chronic diseases. Transcription factors and interactions with the microenvironment determine the fate of T cells during prolonged activation. The efficacy of immune checkpoint blockade relies heavily on the presence and functionality of Tex-prog cells outside the tumor. Future improvements in immunotherapy efficacy are contingent on further research into the molecular mechanisms of exhausted cell differentiation and the development of approaches to the production of more robust and effective effector T cells. Understanding the complex regulation of exhaustion opens up new opportunities for overcoming immune resistance and enhancing the clinical efficacy of adoptive cell therapy and immune checkpoint blockade.

## Abbreviations

AP-1: activator protein 1
BATF: basic leucine zipper transcription factor, ATF-like
Bcl6: B cell lymphoma 6 transcription factor
Blimp-1: B lymphocyte-induced maturation protein 1
BTG1: B cell translocation gene 1
CAR-T: chimeric antigen receptor T cells
CTLA-4: cytotoxic T lymphocyte-associated protein 4
EOMES: Eomesodermin
ETS1: transcription factor ETS-1
Ex-Pre: early precursor exhausted T cells
FOXO1: forkhead box O1
ICB: immune checkpoint blockade
ID3: inhibitor of DNA binding 3
IKAROS: transcription factor Ikaros
IL-2, IL-15, and IL-21: interleukin-2, interleukin-15, and interleukin-21, respectively
IRF4: interferon regulatory factor 4
ITIM: immunoreceptor tyrosine-based inhibitory motif
ITSM: immunoreceptor tyrosine-based switch motif
LAG-3: lymphocyte-activation gene 3
LCMV: lymphocytic choriomeningitis virus
MPEC: memory precursor effector cells
mTOR: mammalian target of rapamycin
MYB: MYB proto-oncogene, transcription factor
NFAT: nuclear factor of activated T cells
NSCLC: non-small cell lung cancer
PD-1: programmed cell death protein 1
PD-L1: programmed death-ligand 1
RBPJ: recombination signal binding protein for immunoglobulin kappa J region
SLEC: short-lived effector cells
STAT5: signal transducer and activator of transcription 5
T-bet: T-box expressed in T cells
TCF1: T cell-specific transcription factor 1
TCR: T cell receptor
TEM: effector memory T cells
TEMRA: effector memory T cells re-expressing CD45RA
Tex: exhausted T cells
Tex-int: intermediate exhausted T cells
Tex-KLR: killer lectin-like receptor-expressing exhausted T cells
Tex-prog: exhausted progenitor T cells
Tex-term: terminally exhausted T cells
Tfh: T follicular helper cells
TIGIT: T cell immunoreceptor with Ig and ITIM domains
TIM-3: T cell immunoglobulin and mucin-domain containing-3
TIL: tumor-infiltrating lymphocytes
TLS: tertiary lymphoid structures
TOX: thymocyte selection-associated HMG box protein
ZEB2: zinc finger E-box binding homeobox 2
